# Prolactin is an Endogenous Antioxidant Factor in Astrocytes That Limits Oxidative Stress-Induced Astrocytic Cell Death via the STAT3/NRF2 Signaling Pathway

**DOI:** 10.1007/s11064-024-04147-3

**Published:** 2024-05-17

**Authors:** Miriam Ulloa, Fernando Macías, Carmen Clapp, Gonzalo Martínez de la Escalera, Edith Arnold

**Affiliations:** 1 Instituto de Neurobiología, Universidad Nacional Autónoma de México, Campus UNAM-Juriquilla, 76230 Querétaro, México; 2 Posgrado en Ciencias Biológicas, Universidad Nacional Autónoma de México, Ciudad Universitaria, 04510 Mexico City, México; 3 CONAHCYT-Universidad Nacional Autónoma de México, Campus UNAM-Juriquilla, Querétaro, México

**Keywords:** Prolactin, Astrocytes, STAT3, NRF2, Antioxidant, Oxidative stress

## Abstract

Oxidative stress-induced death of neurons and astrocytes contributes to the pathogenesis of numerous neurodegenerative diseases. While significant progress has been made in identifying neuroprotective molecules against neuronal oxidative damage, little is known about their counterparts for astrocytes. Prolactin (PRL), a hormone known to stimulate astroglial proliferation, viability, and cytokine expression, exhibits antioxidant effects in neurons. However, its role in protecting astrocytes from oxidative stress remains unexplored. Here, we investigated the effect of PRL against hydrogen peroxide (H_2_O_2_)-induced oxidative insult in primary cortical astrocyte cultures. Incubation of astrocytes with PRL led to increased enzymatic activity of superoxide dismutase (SOD) and glutathione peroxidase (GPX), resulting in higher total antioxidant capacity. Concomitantly, PRL prevented H_2_O_2_-induced cell death, reactive oxygen species accumulation, and protein and lipid oxidation. The protective effect of PRL upon H_2_O_2_-induced cell death can be explained by the activation of both signal transducer and activator of transcription 3 (STAT3) and NFE2 like bZIP transcription factor 2 (NRF2) transduction cascades. We demonstrated that PRL induced nuclear translocation and transcriptional upregulation of *Nrf2*, concurrently with the transcriptional upregulation of the NRF2-dependent genes heme oxygenase 1, *Sod1, Sod2*, and *Gpx1*. Pharmacological blockade of STAT3 suppressed PRL-induced transcriptional upregulation of *Nrf2*, *Sod1* and *Gpx1 mRNA,* and SOD and GPX activities. Furthermore, genetic ablation of the PRL receptor increased astroglial susceptibility to H_2_O_2_-induced cell death and superoxide accumulation, while diminishing their intrinsic antioxidant capacity. Overall, these findings unveil PRL as a potent antioxidant hormone that protects astrocytes from oxidative insult, which may contribute to brain neuroprotection.

## Introduction

Increased generation of reactive oxygen species (ROS), known as oxidative stress, mediates neuronal loss in the majority of neurodegenerative disorders [1]. Oxidative stress contributes to neurodegeneration by altering biomolecular function. Elevated levels of ROS cause protein, DNA, and RNA oxidation, as well as lipid peroxidation, leading to changes in cellular function and cell death [2]. Consequently, oxidative stress has been considered a viable target for therapeutic intervention in the treatment of neurodegeneration. Although studies in animal models of neurodegenerative disorders treated with broad-spectrum ROS scavengers, such as vitamin C and E, have shown significant neuroprotection and cognitive improvements [3, 4], clinical trials with the same broad-spectrum ROS scavengers have not proven effective as a therapy for neurodegenerative disorders of the central nervous system (CNS) [5]. In this regard, a better understanding of the fine-tuned regulation of CNS antioxidant defenses should allow researchers to identify novel targets for more specific therapeutic interventions.

Astrocytes are the first line of antioxidant defense against neurodegenerative disorders, as they express a profuse battery of antioxidant enzymes, including superoxide dismutases (SODs) [6], glutathione peroxidases (GPXs) [6–8], peroxiredoxins [9], and NAD(P)H dehydrogenase [quinone] 1 [10], and are highly enriched in reduced glutathione (GSH), a non-enzymatic ROS scavenger [11]. When CNS homeostasis is disturbed because of a redox imbalance, astrocytes become reactive, responding through up-regulation of antioxidant enzymes and GSH production for protection against the toxic potential of oxidants [12, 13]. Therefore, the identification of molecules that trigger antioxidant mechanisms in astrocytes may lead to the development of potential therapeutic strategies for oxidative stress-induced neurodegeneration.

Astrocytes are direct targets of hormone action in the CNS via receptors expressed on their surface. In this regard, prolactin (PRL), a peptide hormone primarily secreted by the anterior pituitary gland and initially discovered for its effects on lactation, can exert actions in astrocytes under physiological and pathological conditions [14–17]. PRL stimulates the proliferation, viability, and cytokine expression of astrocytes in culture [18, 19] and regulates reactive astrogliosis and the inflammatory response after brain trauma and kainic acid-induced excitotoxicity [16, 19]. Moreover, our previous studies showed that PRL is neuroprotective in the retina under oxidative stress-related conditions, such as exposure to constant bright light and aging [20, 21], and increases the levels of endogenous antioxidants in retinal pigment epithelium (RPE) cells in culture [22]. PRL actions in the CNS are mainly mediated by the long isoform of the PRL receptor which belongs to the class 1 cytokine receptor superfamily and is constitutive associated with the Janus kinases (JAK)/signal transducers and activators of transcription (STAT) pathway [23]. Three members of the STAT proteins’ family have been reported to be activated by phosphorylation mediated by the PRL receptor, namely STAT1, STAT3 and STAT5 [24]. STAT3 is known to play an important role in the regulation of oxidative stress responses in the CNS [25]. Therefore, the question of whether PRL promotes the antioxidant capacity of astrocytes is relevant and could lead to novel therapeutic approaches. In the present study, we used pharmacological and genetic strategies for PRL receptor (PRLR) signaling blockage in primary cultured astrocytes to explore the proposed antioxidant regulation of PRL in astrocytes.

## Materials and Methods

### Animals

Wistar rats were purchased from Charles River Laboratories (USA) and C57BL/6 mice wild-type and heterozygous for the PRLR were purchased from The Jackson Laboratory (USA). Rats and mice colonies were expanded and maintained for several generations in our vivarium. Pregnant Wistar rats or C57BL/6 mice heterozygous for PRLR were separated and housed individually until term. All animals were fed ad libitum and reared in normal cyclic light conditions (12 h light; 12 h dark). On the day of birth (P0), mouse pups were genotyped for *Prlr* and neomycin sequence by DNA isolation from tail tips and amplification using the Azura mouse genotyping kit (Raynham, MA, USA), followed by agarose gel electrophoresis according to the manufacturer’s instructions. Following genotyping, mice were separated into wild-type and PRLR knock-out litters. Briefly, two newborn rat pups (P1) or four mouse (P0) pups of each genotype (both sexes) were anesthetized on ice to minimize animal suffering and then euthanized by decapitation. All animal procedures were done in accordance with the National Institutes of Health Guide for the Care and Use of Laboratory Animals. The Bioethics Committee of the Institute of Neurobiology at the National University of Mexico approved all animal experiments (NOM-062-ZOO-1999).

### Primary Astrocyte Culture

Briefly, primary astrocyte cell cultures were established from brain cortices obtained from newborn Wistar rats and wild-type (*Prlr*^+*/*+^) and PRLR-null (*Prlr*^*−/−*^) C57BL6 mice according to the method previously described [26], with some modifications. The cerebral cortices were dissected under sterile conditions into ice-cold Hank’s Balanced Salt Solution (HBSS, cat. no. 14175095; Thermo Fisher Scientific, NY, USA) and the meninges were removed. Two rat cortices or four mouse meninges-free cortices were pooled, mechanically dissociated, and trypsinized (0.05% in HBSS; cat. no. 27250018; Thermo Fisher Scientific, NY, USA) for 20 min, then centrifuged at 4000 rpm for 5 min. The supernatant was discarded and the pellet resuspended in dulbecco’s modified eagle medium (DMEM, cat. no. L0103; Biowest, MO, USA) containing 10% fetal bovine serum (cat. no. 26140079; Thermo Fisher Scientific, NY, USA) and 1% penicillin/streptomycin (100 units/ml; 100 μg/ml; cat. no. 15140122; Thermo Fisher Scientific, NY, USA). The cell suspension was seeded in 75 cm^2^ flasks coated with poly-D-lysine (cat. no. P6407; Sigma–Aldrich, MO, USA). The cultures were incubated at 37 °C in a humidified 5% CO_2_ atmosphere. The total volume of the culture medium was changed every 4 days. Cells were cultured until they reached confluence (12–13 days); then, to remove any contamination from other cell types, the cultures were treated with 10 μM Cytosine β-D-arabinofuranoside (AraC; cat. no. C1768; Sigma–Aldrich, MO, USA) and shaken in an orbital shaker at 240 rpm for 6 h at 37 °C. The attached cells were cultured for 4 more days and shaken again. Based on anticipated future experiments, cells were seeded at a density of 50,000 cells/cm^2^ onto culture plates of varying sizes. The cultures were incubated under standard conditions for 7 more days until they reached confluence. Astrocyte purity was assessed by staining fixed cell cultures with an anti-glial fibrillary acidic protein (GFAP) antibody (1:1000 dilution; cat. no. AB5804, RRID: AB_2109645; EMD Millipore, MA, USA), revealed with an Alexa Fluor 555 fluorescent secondary antibody (1:5000 dilution; ab150078, RRID: AB_2722519; Abcam, Cambridge, UK). Nuclei were stained with DAPI. Images were recorded on a confocal fluorescence microscope (LSM 780; Zeiss, Oberkochen, GER). Approximately 98% of cells were GFAP-positive in both rat and mouse cultures.

### Experimental Design and Cell Treatments

Considering previous findings that astrocyte cultures from rats and mice exhibit different functional responses [27, 28], and to elucidate the specific and endogenous response of astrocytes to PRL, experiments were conducted using primary astrocytes derived from either neonate rats or *Prlr*^+*/*+^ and *Prlr*^*−/−*^ mice during their first passage in culture. Cells were then treated with one of the following: vehicle (PBS, pH 7.5), ovine PRL (cat. no. L6520; Sigma–Aldrich, MO, USA) or a combination of PRL and STAT3 inhibitor (S3I-201; cat. no. 573102; Merck–Calbiochem, CA, USA) for 24 h. This was followed by a 30 min H_2_O_2_ (cat. no. H3410; Sigma–Aldrich, MO, USA) treatment to investigate the effects of PRL on cell viability, LDH release, ROS generation, protein oxidation, lipid peroxidation, antioxidant capacity, and antioxidant enzyme activity. Separate cultures without H_2_O_2_ treatment were used for *Nrf2* and antioxidant enzymes transcriptional analysis. Additional cultures were treated with vehicle or PRL alone for a shorter duration to assess STAT3 phosphorylation and NRF2 nuclear translocation. PRL and H_2_O_2_ concentrations used were determined experimentally with dose-response curves. S3I-201 was used at 100 μM, a concentration previously shown to prevent STAT3 phosphorylation in vitro [29, 30].

### RNA Isolation and cDNA Synthesis

Total RNA was isolated from primary astrocytes grown and treated in 6-well plate using RNeasy Mini Kit (cat. no. 74106; Qiagen, CA, USA) according to the manufacturer’s instructions. RNA quantification was performed using a Nanodrop ND-1000 spectrophotometer (Thermo Fisher Scientific, DE, USA). cDNA synthesis was carried out from 1 μg of RNA using the high-capacity cDNA reverse transcription kit (cat, no, 4368813; Applied Biosystems-Thermo Fisher Scientific, MA, USA), according to the manufacturer’s instructions. This cDNA served as the template for further polymerase chain reaction (PCR) analysis.

### Quantitative PCR

Primers for real-time PCR amplification of the *Cat*, *Sod1*, *Gpx1*, *Hmox1*, *Nrf2*, and *Sod2* mRNA transcripts were designed using the NCBI primer-designing tool, and their amplification efficiency was evaluated. For *Prlr* and *Hprt* primers, we used previously designed primers reported in our work [21] (Table 1). PCR products were detected and quantified with Maxima SYBR Green/ROX qPCR Master Mix (cat. no. K0223; Thermo Fisher Scientific, Vilnius, LTU) in a 10 μl final reaction volume containing template and 0.5 μM of each primer. Amplification was performed in the CFX96 real-time PCR detection system (Bio-Rad Laboratories, CA, USA) and conditions were 15 s at 95 °C, 30 s at the primer pair-annealing temperature, and 30 s at 72 °C for 40 cycles. The PCR data were analyzed by the 2^−ΔΔCt^ method, and cycle thresholds were normalized to the housekeeping gene hypoxanthine–guanine phosphoribosyl transferase (*Hprt*).

**Table 1:** Primers used for real-time PCR

mRNA	NCBI accession number		Sequence	Amplicon size (bp)	Primer efficiency (%) or [Ref.]
*Cat*	NM_012520.2	F	ACCCACAAACTCACCTGAAG	142	99.2
		R	TGTGAGCCATAGCCATTCATG		
*Gpx1*	NM_030826.4	F	CTCGGTTTCCCGTGCAAT	76	98.6
		R	CATACTTGAGGGAATTCAGAATCTCTT		
*Hmox1*	NM_012580.2	F	CCTGCTAGCCTGGTTCAAG	145	97.6
		R	CATAAATTCCCACTGCCACG		
*Nrf2*	NM_031789.2	F	CCACATTCCCAAACAAGATGC	132	97.8
		R	GTGAAGACTGAGCTCTCAACG		
*Sod1*	NM_017050.1	F	GGTGTCAGGACAGATTACAGG	130	94.5
		R	ACCGCCATGTTTCTTAGAGTG		
*Sod2*	NM_017051.2	F	TCAGGACCCACTGCAAGGA	66	123.2
		R	GCGTGCTCCCACACATCA		
*Prlr*	NM_012630.2	F	CCAGGAGAGTTCCGTTGAAATG	153	[21]
		R	GGTGGAAAGATGCAGGTCAT		
*Hprt*	NM_012583.2	F	GACCGGTTCTGTCATGTCG	61	[21]
		R	ACCTGGTTCATCATCACTAATCAC		

### MTT Assay

Cell viability was measured in primary astrocytes that were grown and treated in 96-well plate using the tetrazolium salt, 3-(4,5-dimethylthiazol-2-yl)-2,5-diphenyltetrazolium bromide (MTT) assay (cat. no. M5655; Sigma–Aldrich, MO, USA). Ten microliters of MTT solution (5 mg/mL in PBS) were added aseptically to the treated cells in each of the wells, followed by incubation at 37 °C for 3 h. The media was aspirated and 10% sodium dodecyl sulphate (SDS) in 0.01 M HCl was added to dissolve the insoluble formazan crystals. The absorbance of colored solutions was quantified by a spectrophotometer with an excitation wavelength of 570 nm (Varioskan Flash, Thermo Fisher Scientific, Vantaa, FIN).

### LDH Cytotoxicity Assay

The cytotoxicity of H_2_O_2_ was assessed by the measurement of LDH release into the culture medium using the CytoTox 96® non-radioactive cytotoxicity assay kit (cat. no. G1780; Promega, WI, USA), according to the manufacturer’s instructions. Aliquots of 50 μl of the conditioned medium of astrocytes grown and treated in 96-well plate, were transferred to a new 96-well culture plate and mixed with the LDH assay kit reagent for 30 min. Stop solution was added to each well and the absorbance of colored solutions was quantified by a spectrophotometer with an excitation wavelength of 492 nm (Varioskan Flash, Thermo Fisher Scientific, Vantaa, FIN). Results were expressed as percent of total LDH release, achieved by treating non-incubated cells with lysis solution prior to the assay for maximum LDH release.

### Measurement of Anion Superoxide

Superoxide production in astrocytes grown and treated in 96-well plate was measured using dihydroethidium (DHE; cat. no. D23107; Thermo Fisher Scientific, MA, USA) as previously described [6]. The medium was removed, and the wells were washed once with 200 μl of PBS. H_2_O_2_ was added in 200 μl of PBS to the appropriate rows and incubated for 30 min. H_2_O_2_ was removed, and the wells were washed once with PBS; 10 μM DHE in PBS was then applied and fluorescence was read at Ex = 530 nm and Em = 595 nm over a 30 min period.

### Measurement of Reactive Oxygen Species

ROS production in astrocytes grown and treated in 96-well plate was measured using 2′,7′-dichlorodihydrofluorescein diacetate (DCF-DA; cat. no. D6883; Sigma–Aldrich, MO, USA) as previously described [6]. The medium was removed and wells were washed twice with 100 μl of PBS. A total of 20 μg/ml DCF-DA was added to the wells in 100 μl of PBS and incubated for 30 min. The wells were then washed twice with PBS and H_2_O_2_ added in 100 μl PBS. DCF fluorescence was read every 15 min for 90 min at Ex = 485 nm and Em = 530 nm.

### Measurement of Total Antioxidant Capacity

Astrocytes grown and treated in 96-well plate were harvested in ice-cold PBS and centrifuged at 2000 rpm for 10 min at 4 °C. The cell pellet was sonicated in 1 ml of buffer (5 mM potassium phosphate, pH 7.4, containing 0.9% sodium chloride and 0.1% glucose) for 5 s and centrifuged at 13,200 rpm for 20 min at 4 °C. Total antioxidant capacity was assessed in the supernatants by ABTS assay according to the manufacturer’s instructions (cat. no. 709001; Cayman Chemical, MI, USA). The antioxidant capacity of the samples was compared with that of a water-soluble tocopherol (Trolox) and was calculated by extrapolating the value from the most linear part of the Trolox standard curve and expressed as the millimolar Trolox equivalent/mg of protein.

### Measurement of Carbonyl Protein Content

Protein oxidation was determined using a modified method described by Levine [31]. Astrocytes grown and treated in 6-well plate were scraped off, homogenized in 100 μl ice-cold PBS, and centrifuged at 2900 rpm 10 min at 4 °C. Supernatant protein concentration was estimated using the Bradford method. Briefly, 100 μl of 2,4-dinitrophenylhydrazine (DNPH; 10 nM in 2 M HCl; cat. no. D199303; Sigma–Aldrich, MO, USA) was added to 50 μl of supernatant sample and incubated at room temperature for 1 h in darkness, vortexing every 15 min. Afterward, 125 μl of 20% trichloroacetic acid (TCA; cat. no. T6399; Sigma–Aldrich, MO, USA) was added to the mixture and centrifuged at 13,200 rpm for 3 min. The supernatant was discarded, and the pellet was resuspended in 200 μl of an ethanol-ethyl acetate (1:1) mix and centrifuged again at 20,000 g for 3 min. Finally, the pellet was resuspended in 200 μl of 6 M guanidine (cat. no. G3272; Sigma–Aldrich, MO, USA) dissolved in 20 mM K_2_PO_4_, pH 2.3 (cat. no. P5629; Sigma–Aldrich, MO, USA). The absorbance of the supernatant was measured at 370 nm. Carbonyl protein content was quantified using an extinction coefficient of 2.2 × 10^5^ M^−1^ cm^−1^ and expressed as nanomole carbonyl per milligram of protein.

### Measurement of Total Reduced Thiol (SH) Content

Thiol groups concentration was quantified using the Ellman’s reagent (2-nitrobenzoic acid, DTNB) by the method previously described with some modifications [32]. Primary astrocytes grown and treated in 6-well plates were scraped off and homogenized in 100 μl ice-cold PBS, and the suspensions were centrifuged at 2900 rpm g for 10 min at 4 °C. Supernatant protein was estimated using the Bradford method. Briefly, 50 μl ethylenediaminetetraacetic (EDTA) (cat. no. E5134; Sigma–Aldrich, MO, USA) and 7.5 μl DTNB reagent (10 nM; cat. no. D218200; Sigma–Aldrich, MO, USA) were added to a 25 μl supernatant sample. The mixture was incubated at room temperature for 30 min and centrifuged at 10,400 rpm for 2 min. Afterward, the absorbance of the supernatant was read at 412 nm. Total thiol concentration was calculated using an extinction coefficient of 1.41 × 10^5^ M^−1^ cm^−1^ and expressed as nanomole per milligram of protein.

### TBARS Assay

Lipid peroxidation products were quantified by measuring thiobarbituric acid reactive substances (TBARS) by the method previously described [33]. Primary astrocytes grown and treated in 6-well plates were scraped off and homogenized in 100 μl ice-cold PBS, and the suspensions were centrifuged at 2900 rpm for 5 min at 4 °C. Supernatant protein was estimated using the Bradford method. Briefly, 75 μl of supernatant and 75 μl of TRIS–HCl (20 nM, pH 7.4) were incubated at 37 °C for 2 h. After incubation, 150 μl of 10% TCA was added and centrifuged at 3300 rpm for 10 min at 4 °C. Then, 300 μl of supernatant was transferred to glass tubes and 300 μl of 0.5% (w/v) thiobarbituric acid (cat. no. IC19028480, VWR, PA, USA) was added, and the tubes were kept in boiling water for 10 min. After cooling to room temperature, the absorbance of the supernatant at 532 nM was determined. TBARS were quantified using an extinction coefficient of 1.56 × 10^5^ M^−1^ cm^−1^ and expressed as nanomoles of malondialdehyde (MDA) per milligram of protein.

### Western Blot

Primary astrocytes grown and treated in 100 mm dishes were scraped off with 1 ml of ice-cold PBS. After centrifugation at 13,200 rpm for 5 min at 4 °C, the cell pellet was resuspended in 50 μl of RIPA lysis buffer (1 M Tris HCl, pH 7.5; 0.2 M EGTA, pH 7.5; 0.2 M EDTA, pH 7.5; 1% Nonidet P-40; 0.1 M Na_3_VO_4_; 0.05 M NaF; 5 mM Na_4_P_2_O_7_; 0.26 M sucrose) supplemented with a protease inhibitor cocktail (cOmplete; cat. no. 11697498001; Roche, Mannheim, GER), incubated for 30 min on ice, and centrifuged at 12,000 rpm for 20 min at 4 °C. The supernatant was kept at 70 °C until analysis. The protein concentration of the sample was determined according to the Bradford method with bovine serum albumin (BSA) as a standard. Samples containing equal amounts of protein (5, 10, 20 or 30 μg) and Laemmli sample buffer (10% SDS, 20% glycerol, 0.5% bromophenol blue, 0.5 M Tris HCl, β-mercaptoethanol) were heated at 99 °C for 1 min. After separation on SDS-polyacrylamide gels (10%), proteins were transferred to nitrocellulose membranes (cat. no. 1620112; Bio-Rad Laboratories, CA, USA). Non-specific binding was inhibited by incubation in TBST (Tris-buffered saline with 0.1% Tween 20 detergent) containing 5% non-fat milk (cat. no. 1706404; Bio-Rad Laboratories, CA, USA) for 1 h at room temperature. The blots were then incubated overnight at 4 °C with polyclonal antibodies against phospho-STAT3 (1:2000 dilution; cat. no. 9145, RRID:AB_2491009; Cell Signaling Technology, MA, USA), STAT3 (1:350 dilution; cat. no. sc-483, RRID:AB_632441; Santa Cruz Biotechnology, TX, USA), GPX1(1:100 dilution; cat. no. bs-3882R, RRID:AB_10857071; Bioss Antibodies, MA, USA) and β-tubulin (1:500 dilution; cat. no. ab6046, RRID:AB_2210370; Abcam, Cambridge, UK); or monoclonal antibodies against SOD1 (1:700 dilution; cat. no. sc-101523, RRID:AB_2191632; Santa Cruz Biotechnology, TX, USA) and SOD2 (1:100 dilution; cat. no. sc-133134, RRID:AB_2191814; Santa Cruz Biotechnology, TX, USA). The blots were then washed with TBST and incubated at room temperature for 1 h with an appropriate horseradish peroxidase-conjugated antibody (1:5000 dilution; cat. no. 111035144, RRID:AB_2307391; Jackson ImmunoResearch Inc., PA, USA) or alkaline phosphatase-conjugated secondary (1:5000 dilution; cat. no. 111055003, RRID:AB_2337947, cat. no. 115055003, RRID:AB_2338528; Jackson ImmunoResearch Inc., PA, USA). All antibodies were diluted in TBST containing 0.5% BSA. After washing, the secondary antibodies were detected digitally with a Fluor E system (Protein Simple, CA, USA). To evaluate the results of the Western blot analysis, each band was quantified by densitometry using Image J version 1.52a (National Institutes of Health, USA). The integrated optical density of each band was normalized to β-tubulin.

### Antioxidant Enzyme Activity Assays

Catalase, GPX, and SOD enzymatic activity in primary astrocytes was determined with commercial assays (cat. no. 707002, 703102, 706002; Cayman Chemical, MI, USA). Astrocytes grown and treated in 6-well plates were scraped off and homogenized in ice-cold buffer. Samples containing equal amounts of protein of each treatment group were assayed following the manufacturer’s instructions.

### Immunocytochemistry and Image Analysis

Primary astrocytes were seeded and treated on poly-D-lysine-coated glass coverslips. Cells were fixed in 4% paraformaldehyde and 4% sucrose in PBS (pH 7.4) for 10 min at room temperature, washed three times in PBS, and then permeabilized with Triton X-100 (cat. no. X198-07; Avantor, PA, USA) for 10 min at room temperature. Next, cells were blocked by incubation with PBS containing 10% normal goat serum (cat. no. 10000C; Gibco-Thermo Fisher Scientific, NY, USA) for 1 h at room temperature. Incubation with the primary polyclonal antibody anti-NRF2 (1:100 dilution; cat. no. ab31163, RRID:AB_881705; Abcam, Cambridge, UK) was performed overnight at 4 °C. Next, cells were washed and incubated for 1 h at room temperature with the secondary antibody Alexa Fluor 555 goat anti-rabbit (1:1000 dilution; cat. no. ab150078, RRID:AB_2722519; Abcam, Cambridge, UK). Nuclei were counterstained with Sytox green (cat. no. S7020; Thermo Fisher Scientific, OR, USA). Finally, cells were mounted onto glass slides with Vectashield (cat. no. H-1000-10; Vector Laboratories, CA, USA) mounting medium. All images were captured and digitized using a Zeiss LSM 780 confocal microscope (Carl Zeiss, Oberkochen, GER). Quantitative analysis of mean integrated density of NRF2 fluorescence signal in nuclei were performed using ImageJ 1.51 software. Images were acquired using a z-stack of seven to ten images from a microscopic field. The mean integrated fluorescence density in nuclei was quantified from ~ 80 to 100 cells/field of four independent primary astrocyte cultures. Maximum intensity projection was used to form a 2D image.

### Statistics

Graphs and statistical analyses were performed using Prism v.10 software (GraphPad Software, CA, USA). The data normality was tested beforehand using a Shapiro–Wilk test. Differences between groups were determined by one-way ANOVA followed by the Tukey’s test or Kruskal–Wallis’s test followed by Dunn’s test for multiple comparisons or using a two-tailed Student’s t-test. All data are reported as mean ± S.E.M. The threshold for significance was set at *p* < 0.05.

## Results

### PRL Prevents H_2_O_2_-Induced Death of Rat Astrocytes

**Fig. 1:**
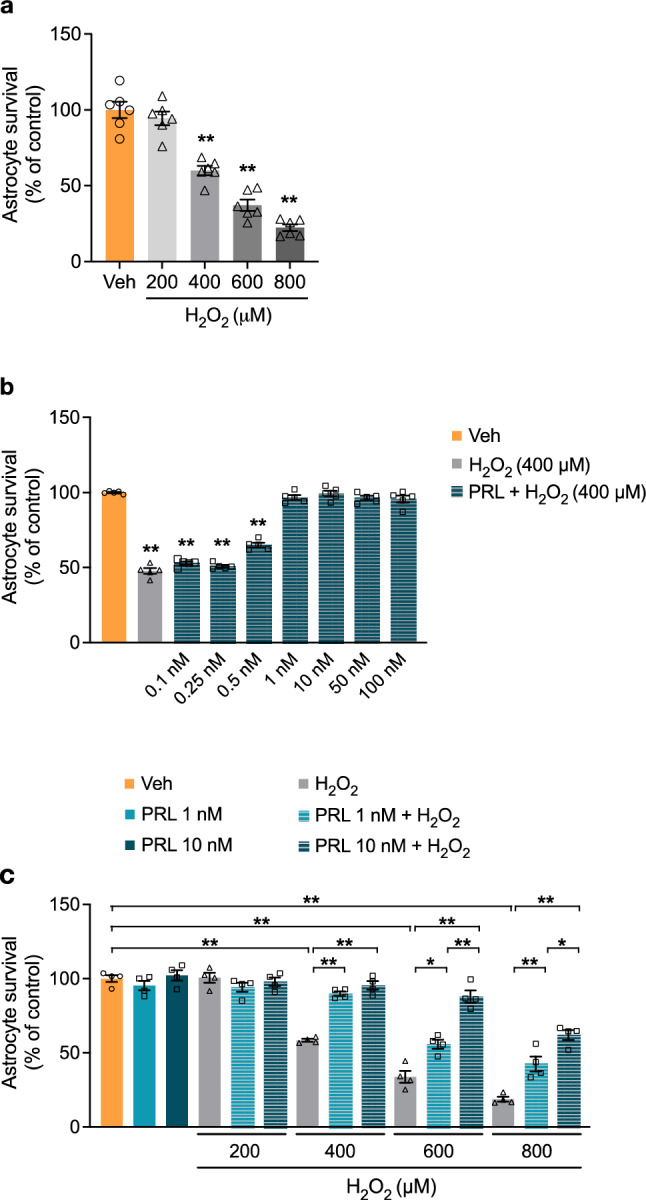
Effect of PRL on H_2_O_2_ toxicity in rat astrocytes. **a** Rat cortical astrocytes were incubated for 3 h in the absence or presence of increasing concentrations (200–800 μM) of hydrogen peroxide (H_2_O_2_). Cell viability was quantified by MTT assay, and the results were normalized to the control with vehicle. Data are means ± SEM of six independent primary cultures (n = 6) carried out in triplicate. **b** Rat cortical astrocytes were pre-incubated for 24 h in the absence or presence of increasing concentrations (1–100 nM) of prolactin (PRL), then 400 μM H_2_O_2_ or vehicle was added and incubated for 3 h. Cell viability was quantified by MTT assay, and the results were normalized to the control with vehicle. Data are means ± SEM of five independent primary cultures (n = 5) carried out in triplicate. **c** Rat cortical astrocytes were pre-incubated for 24 h in the absence or presence of 1 or 10 nM PRL, then 400 μM H_2_O_2_ or vehicle was added and incubated for 3 h. Cell viability was quantified by MTT assay, and the results were normalized to the control with vehicle. Data are means ± SEM of four independent primary cultures (n = 4) carried out in triplicate. One-way ANOVA followed by Tukey’s test; *p < 0.05, **p < 0.001 versus vehicle or indicated group

To mimic the excess ROS production observed in brain injuries and neurodegenerative diseases [34, 35], treatments with micromolar concentrations of H_2_O_2_ (typically ranging from 200 to 500 μM) are a common method to induce oxidative stress in astroglia cells [36–41]. The effect of H_2_O_2_ on the viability of rat astrocytes was tested using an MTT assay to measure metabolic activity as an indicator of cell viability. As expected, treatment of astrocytes for 30 min with increasing concentrations of H_2_O_2_ (0–800 μM) caused a concentration-dependent decrease in cell viability (Fig. 1a). When astrocytes were incubated with 400 μM H_2_O_2_, cell viability was almost halved compared to the non-treated control level (60.03 ± 3.008% vs. 100 ± 5.349%; F_(4, 25)_ = 76.24, *p* = 0.000001; Fig. 1a). Therefore, we selected 400 μM H_2_O_2_ as a paradigm for inducing oxidative stress in subsequent experiments to test the effect of PRL. Pretreatment for 24 h with PRL significantly inhibited H_2_O_2_-induced cell death over a concentration range of 1–100 nM (F_(8,36)_ = 232.2; Fig. 1b). Then, the cytoprotective effect of PRL was investigated in correlation with H_2_O_2_ concentration. Pretreatment with 1 nM PRL blocked the cytotoxic effect of 400 μM H_2_O_2_ (Fig. 1c) and significantly reduced cell death induced by 600 μM H_2_O_2_ (33.82 ± 4.01% vs. 55.78 ± 3.01%; F_(14, 45)_ = 77.70, *p* = 0.00104; Fig. 1c) and 800 μM H_2_O_2_ (18.75 ± 1.63% vs. 42.47 ± 5.04%; *p* = 0.0003; Fig. 1c); meanwhile, 10 nM PRL blocked cell death induced by both 400 and 600 μM H_2_O_2_ and greatly reduced cell death induced by 800 μM H_2_O_2_ (18.75 ± 1.63% vs. 60.416 ± 3.35%; *p* < 0.000001; Fig. 1c). On the basis of these results, we chose a PRL concentration of 10 nM for the following experiments.

### STAT3 Activation is Involved in PRL-Mediated Protection From H_2_O_2_-Induced Cell Death

**Fig. 2:**
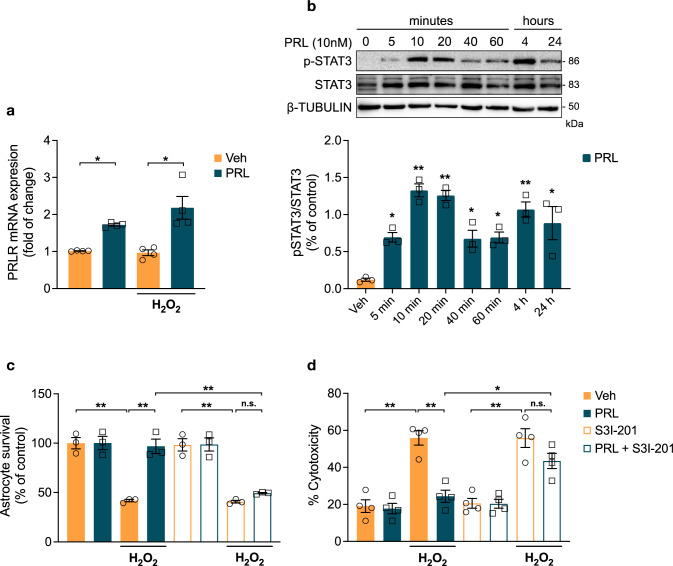
Involvement of STAT3 in the protective effect of PRL on rat astrocytes. **a** Rat cortical astrocytes were pre-incubated for 24 h in the absence or presence of 10 nM prolactin (PRL), then 400 μM hydrogen peroxide (H_2_O_2_) or vehicle (Veh) was added and incubated for 3 h. Prolactin receptor (*Prlr*) mRNA levels were measured by quantitative RT-PCR. Data were normalized using the *Hprt* housekeeping gene as an internal control. Data are means ± SEM of four independent experiments (n = 4). **b** Effect of PRL on STAT3 phosphorylation in rat cortical astrocytes. Active STAT3 was detected by Western blotting in 30 μg of astrocyte lysate using an antibody against phosphorylated STAT3 (pSTAT3) and quantified by using total STAT3 and β-tubulin as internal controls. Data are means ± SEM of three independent experiments (n = 3). Rat cortical astrocytes were pre-incubated in the absence or presence of 10 nM PRL and/or 100 μM STAT3 inhibitor S3I-201 for 24 h, then 400 μM H_2_O_2_ or vehicle was added and incubated for 3 h. **c** Cell viability was quantified by MTT assay. Data are means ± SEM of three independent experiments (n = 3) carried out in triplicate. **d** H_2_O_2_-induced cytotoxicity was quantified by the measurement of LDH release. Results are normalized to the control with vehicle or expressed as percent of total LDH release obtained by treating non incubated cells with lysis solution prior to the assay to induce maximum LDH release, respectively. Data are means ± SEM of four independent experiments (n = 4) carried out in triplicate. One-way ANOVA followed by Tukey’s test; *p < 0.05, **p < 0.001 versus vehicle or between group; n.s. no significant difference

In agreement with previous reports [15, 18], astrocyte cultures prepared from P0-P1 rat cortices expressed the long form of the PRLR (Fig. 2a). Furthermore, since PRL was reported to regulate the expression of its own receptor in several tissues [42, 43], we examined the effect of PRL on *Prlr* expression by quantitative reverse transcription-PCR (RT-qPCR). Treatment with 10 nM PRL for 24 h significantly increased the expression of *Prlr* (1.72 ± 0.059 vs. 1.015 ± 0.012; F_(3,12)_ = 14,42; *p* = 0.0168, Fig. 2a), and this effect was sustained after H_2_O_2_ treatment (2.183 ± 0.612 vs. 0.9675 ± 0.155, *p* = 0.0006). H_2_O_2_ did not modify *Prlr* expression in astrocytes (Fig. 2a). Binding of PRL to PRLR is known to activate the JAK2-STAT pathway [24]. Given the close relationship between STAT3 activation and antioxidant protection in astrocytes [6], we examined the phosphorylation/activation profile of STAT3 in rat primary astrocytes following exposure to 10 nM PRL. As shown in Fig. 2b, PRL induced a sustained increase in STAT3 phosphorylation with a biphasic pattern characterized by an initial peak in the first 10 min (1.326 ± 0.086 vs. 0.1207 ± 0.021; F_(7, 16)_ = 12.62, *p* = 0.000016), followed by a second peak at 4 h (1.068 ± 0.1014 vs. 0.1207 ± 0.021; *p* = 0.0003). Then, STAT3 involvement in the protection of PRL against H_2_O_2_-induced cell death in astrocytes was pharmacologically evaluated using S3I-201, a STAT3 inhibitor that blocks STAT3 phosphorylation, dimerization, DNA binding, and STAT3-dependent transcription [29]. S3I-201, at a concentration (100 μM) previously shown to prevent STAT3 phosphorylation in vitro [29, 30], was added 10 min before PRL treatment. We assessed cell viability by MTT assay and LDH release to the culture medium to investigate whether PRL protected against H_2_O_2_-induced cytotoxicity and loss of membrane integrity. As expected, PRL blocked the H_2_O_2_-induced cell death (58.16 ± 1.23% loss of cell viability compared to control; F_(7, 16)_ = 29.67; *p* = 0.000015; Fig. 2c) and LDH release (55.913 ± 3.88% increase compared to control; F_(7, 24)_ = 22.341; *p* = 0.000003; Fig. 2d). Notably, this protective effect of PRL was abolished by pretreatment with S3I-201 (49.32 ± 0.0602% vs. 96.86 ± 7.29% viability; *p* = 0.0001; Fig. 2c; and 24.4 ± 3.26% vs. 43.48 ± 4.1% LDH release; *p* = 0.016, Fig. 2d). The STAT3 inhibitor alone had no effect on the viability of astrocytes (Fig. 2c, d). The inability of PRL to rescue astrocytes after treatment with S3I-201 indicates that STAT3 activation is required for PRL protection from H_2_O_2_-induced cell death.

### PRL Prevents Oxidative Damage Induced by H_2_O_2_ via STAT3 Activation

**Fig. 3:**
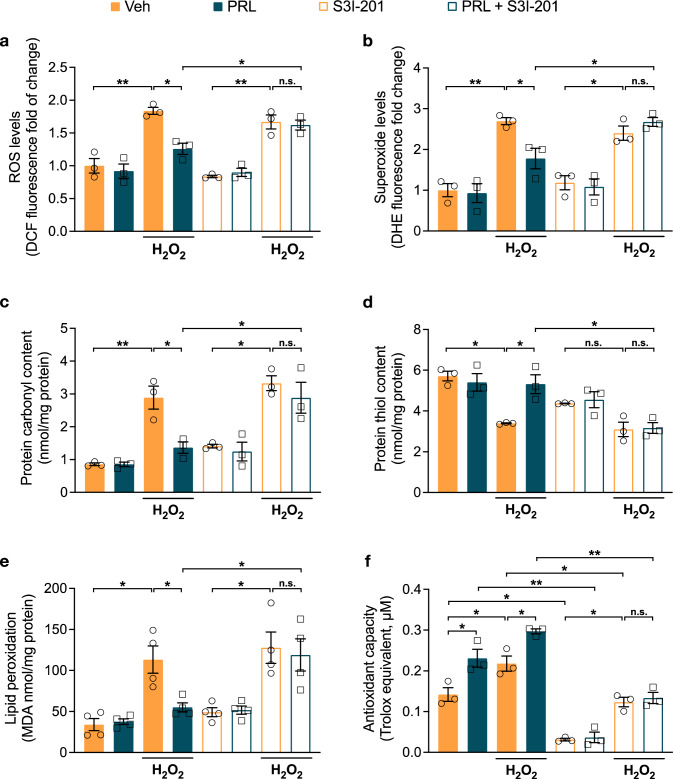
Effect of PRL on H_2_O_2_-induced ROS production and oxidative damage in rat astrocytes. Rat cortical astrocytes were pre-incubated in the absence or presence of 10 nM prolactin (PRL),100 μM STAT3 inhibitor (S3I-201) or both for 24 h, then 400 μM hydrogen peroxide (H_2_O_2_) or vehicle (Veh) was added and incubated for 3 h. **a** Generation of reactive oxygen species (ROS) was quantified using 2′,7′-dichlorodihydrofluorescein diacetate (DCF-DA). Values are expressed as DCF fluorescence after 30 min of incubation. Data are means ± SEM of three independent experiments (n = 3) carried out in triplicate. **b** Generation of superoxide anion was measured using dihydroethidium (DHE). Values are expressed as DHE fluorescence after 30 min of incubation. Data are means ± SEM of three independent experiments (n = 3) carried out in triplicate. **c** Protein oxidation was estimated by measuring the protein carbonyl levels with the DNPH colorimetric assay. The concentration of the protein carbonyls was adjusted to the total protein concentration. Data are means ± SEM of three independent experiments (n = 3) carried out in duplicate. **d** Total sulfhydryl groups were measured by the reaction of free thiols in native proteins with DTNB. The concentration of the free thiols was adjusted to the total protein concentration. Data are means ± SEM of three independent experiments (n = 3) carried out in duplicate. **e** Lipid peroxidation was determined as the increase in malondialdehyde (MDA), a thiobarbituric acid reactive substance (TBARS). The concentration of the MDA was adjusted to the total protein concentration. Data are means ± SEM of four independent experiments (n = 4). **f** Antioxidant capacity was detected by ABTS assay. Data are means ± SEM of three independent experiments (n = 3) carried out in duplicate. One-way ANOVA followed by Tukey’s test; *p < 0.05, **p < 0.001 versus indicated group; n.s. no significant difference

Previous studies demonstrated that H_2_O_2_ induces ROS elevation in cultured astrocytes and that a marked elevation in ROS leads to cell death [39, 44]. Since PRL rescued astrocytes from H_2_O_2_-induced death, we examined the effect of the PRL/PRLR/STAT3 pathway on ROS generation. ROS were measured using DCF-DA, a probe that fluoresces in reaction with H_2_O_2_, hydroxyl radical, nitric oxide, and peroxynitrite but not with superoxide [45]. ROS generation was 1.8-fold higher in H_2_O_2_-treated astrocytes relative to control conditions (1.837 ± 0.054 vs. 1.0 ± 0.111, F_(7, 16)_ = 23.12, *p* = 0.000055; Fig. 3a). PRL significantly reduced the H_2_O_2_-induced elevation in ROS (1.258 ± 0.085 vs. 1.837 ± 0.054, *p* = 0.00305; Fig. 3a). Superoxide levels were measured using the DHE fluorescent probe. Superoxide generation was 2.7-fold higher in H_2_O_2_-treated astrocytes relative to control conditions (2.693 ± 0.085 vs. 1 ± 0.16, F_(7, 16)_ = 18.41, *p* = 0.00012; Fig. 3b). PRL significantly reduced the H_2_O_2_-induced elevation in superoxide (1.77 ± 0.25 vs. 2.693 ± 0.08, *p* = 0.038; Fig. 3b). S3I-201 blocked the PRL-mediated reduction in ROS (1.621 ± 0.074, *p* = 0.0326*;* Fig. 3a) and superoxide levels (2.674 ± 0.1077, *p* = 0.044; Fig. 3b), which is consistent with the role that STAT3 plays in PRL’s protective effect on astrocyte viability. These results indicate that PRL can diminish H_2_O_2_-induced oxidative stress and that STAT3 mediates this effect. Oxidative stress is widely implicated in the oxidation of biomolecules and can greatly affect cell viability. Thus, we assessed the effect of PRL on the oxidative damage of proteins and lipids. Protein oxidation was determined by measuring protein carbonyl and sulfhydryl levels. Astrocytes treated with 400 μM H_2_O_2_ had a significant 3.4-fold increase (2.885 ± 0.3496 vs. 0.8556 ± 0.04103 nmol/mg protein; F_(7, 16)_ = 15.74, *p* = 0.00000441) in carbonyl levels (Fig. 3c) and a 40.6% decrease (3.391 ± 0.02494 vs. 5.711 ± 0.2371 nmol/mg protein; F_(7, 16)_ = 10.98, *p* = 0.00004) in sulfhydryl levels compared to control conditions (Fig. 3d). PRL treatment prevented both the increase of carbonyl groups (17.05 ± 2.182 nmol/mg protein; *p* = 0.0114) and the reduction of sulfhydryl levels (26.6 ± 2.332 nmol/mg protein, *p* = 0.0116) induced by H_2_O_2_ in astrocytes (Fig. 3c, d). Lipid peroxidation was determined by measuring TBARS content expressed as the level of MDA. As shown in Fig. 3e, astrocytes treated with 400 μM H_2_O_2_ had a significant 3.3-fold increase in MDA content as compared to control conditions (113.2 ± 16.57 vs. 34.08 ± 7.389 nmol/mg protein, F_(7, 24)_ = 8.282, *p* = 0.023). PRL treatment prevented MDA increase in H_2_O_2_-treated astrocytes (55.04 ± 5.534 nmol/mg protein, *p* = 0.042, Fig. 3e). Moreover, PRL protection in carbonyl generation (36.05 ± 5.863 vs. 17.05 ± 2.182 nmol/mg protein, *p* = 0.0114, Fig. 3c), sulfhydryl loss (15.82 ± 1.323 vs. 26.6 ± 2.332 nmol/mg protein, *p* = 0.0043, Fig. 3d) and lipid peroxidation (118.9 ± 19.60 vs. 55.04 ± 5.534 MDA nmol/mg protein, *p* = 0.0017, Fig. 3e) was reversed in astrocytes treated with the STAT3 inhibitor (S3I-201), demonstrating that STAT3 is required for PRL antioxidant protection. The STAT3 inhibitor by itself did not affect carbonyl, sulfhydryl, or MDA levels in astrocytes (Fig. 3c–e). These results clearly implicate PRL as an effective trigger of antioxidant responses in H_2_O_2_-treated astrocytes. Thus, we assessed whether PRL induces changes in endogenous antioxidant status by measuring the total antioxidant capacity of astrocytes upon exposure to PRL and/or H_2_O_2_. We observed a significant 1.62-fold increase (0.231 ± 0.022 vs. 0.142 ± 0.017 Trolox equivalent μM, F_(7, 16)_ = 42.087, *p* = 0.008; Fig. 3f) and 1.53-fold increase (0.218 ± 0.019 vs. 0.142 ± 0.017 Trolox equivalent μM, *p* = 0.029; Fig. 3f) in antioxidant capacity in astrocytes treated with PRL or H_2_O_2_, respectively. Moreover, PRL pretreatment induced a further 1.36-fold increase in the antioxidant capacity of H_2_O_2_-treated astrocytes in comparison to the level induced by H_2_O_2_ alone (0.297 ± 0.006 vs. 0.218 ± 0.019 Trolox equivalent μM, *p* = 0.022; Fig. 3f). Notably, the STAT3 inhibitor alone diminished the antioxidant capacity of astrocytes in the absence (0.0311 ± 0.0032 vs. 0.142 ± 0.017 Trolox equivalent μM, *p* = 0.001) or presence of PRL (0.0366 ± 0.013 vs. 0.231 ± 0.022, Trolox equivalent μM, *p* = 0.0000011*;* Fig. 3f) and blocked the increase in the antioxidant capacity observed in PRL-treated astrocytes in response to H_2_O_2_ treatment (0.1336 ± 0.0134 vs. 0.2969 ± 0.0062 Trolox equivalent μM, *p* = 0.000018*;* Fig. 3f). These results suggest that STAT3 is partially responsible for the basal antioxidant activity of astrocytes and fully responsible for the action of PRL. In summary, these data demonstrate that PRL significantly enhances the antioxidant capacity of astrocytes, resulting in a reduction of H_2_O_2_-induced ROS production and protein and lipid oxidation.

### PRL Up-Regulates Antioxidant Enzyme Genes in Astrocytes Through STAT3 Signaling

**Fig. 4:**
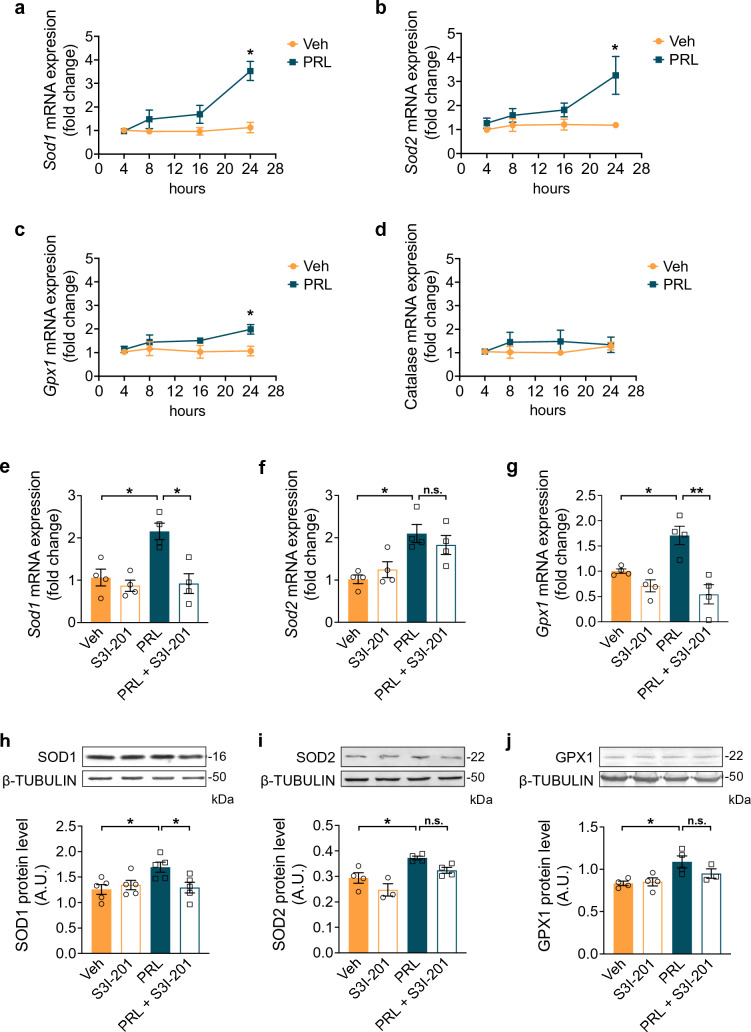
Effect of PRL on transcription of antioxidant genes in rat astrocytes. Rat cortical astrocytes were incubated in the absence or presence of 10 nM prolactin (PRL) for 4, 8, 16 or 24 h, and changes in mRNA levels of **a** superoxide dismutase 1 (*Sod1)*, **b** superoxide dismutase 2 (*Sod2)*, **c** glutathione peroxidase 1 (*Gpx1)*, and **d** catalase were measured by quantitative RT-PCR. Data were initially normalized using the *Hprt* housekeeping gene as an internal control and then to the corresponding gene expression in the 4 h vehicle-treated group (Veh). Data in **a**, **b**, **c** and **d** are means ± SEM of four independent experiments (n = 4). Unpaired two-tailed Student’s t-test. Rat cortical astrocytes were incubated in the absence or presence of either 10 nM PRL, 100 μM STAT3 inhibitor S3I-201 or both for 24 h. mRNA levels of **e** *Sod1*, **f** *Sod2*, and **g** *Gpx1* were measured by quantitative RT-PCR. Data were initially normalized using the *Hprt* housekeeping gene as an internal control and then to the corresponding gene expression in the vehicle-treated group. Data in **e**, **f**, and **g** are means ± SEM of four independent experiments (n = 4). Protein expression of SOD1 (**h**), SOD2 (**i**), and GPX1 (**j**) was detected by Western blotting in 5, 10 or 20 μg of astrocyte lysate, respectively; and quantified by using β-tubulin as loading control. Data are means ± SEM of (**h**, **j**) four (n = 4) or (j) three (n = 3) independent experiments. One-way ANOVA followed by Tukey’s test. *p < 0.05, **p < 0.001 versus vehicle or indicated group. For GPX1 protein, Kruskal–Wallis’s test followed by Dunn’s test

Given that ROS accumulation and oxidative damage are mostly counteracted by antioxidant enzymes, we next assessed the effect of PRL on the mRNA expression of antioxidant enzyme genes including *Sod1*, *Sod2*, *Gpx1*, and catalase. Cultured astrocytes were treated with 10 nM PRL for 4, 8, 16 and 24 h. Exposure to PRL significantly increased *Sod1*, *Sod2*, and *Gpx1* mRNA levels in astrocytes only at 24 h of incubation (Fig. 4a–c). mRNA for *Sod1* increased about 3.15-fold (3.53 ± 0.406 vs. 1.125 ± 0.406, *p* = 0.0021*;* Fig. 4a); for *Sod2*, it increased about 2.75-fold (3.253 ± 0.788 vs. 1.185 ± 0.1084, *p* = 0.0407*;* Fig. 4b); and for *Gpx1*, it increased 1.86-fold (1.988 ± 0.2016 vs. 1.068 ± 0.2034, *p* = 0.0183*;* Fig. 4c). In contrast, catalase mRNA levels did not change at any time (Fig. 4d). We then investigated whether STAT3 could be involved in PRL-induced transcriptional changes by inhibiting STAT3 signaling with S3I-201 before PRL treatment. We found that the PRL-induced increase in the mRNA levels of *Sod1* and *Gpx1* at 24 h of incubation were abrogated when STAT3 signaling was inhibited, whereas *Sod2* expression was not affected (Fig. 4e–g). Consistently, PRL-treated astrocytes showed a significant increase in SOD1 (1.692 ± 0.2193 vs. 1.257 ± 0.2148 A.U., F_(3, 16)_ = 4.202, *p* = 0.0282), SOD2 (0.3723 ± 0.023 vs. 0.2937 ± 0.041 A.U., F_(3, 11)_ = 9.781, *p* = 0.021), and GPX1 (1.088 ± 0.1408 vs. 0.8327 ± 0.051 A.U., F_(3, 11)_ = 5.258, *p* = 0.020*)* protein levels, that were abrogated by STAT3 inhibition in the case of SOD1 (Fig. 4g–i). Taken together, these results indicate that the downstream signaling of PRL via STAT3 causes a robust induction of antioxidant enzyme gene and protein expression in astrocytes.

### PRL Activates the NRF2 Pathway in Astrocytes

**Fig. 5:**
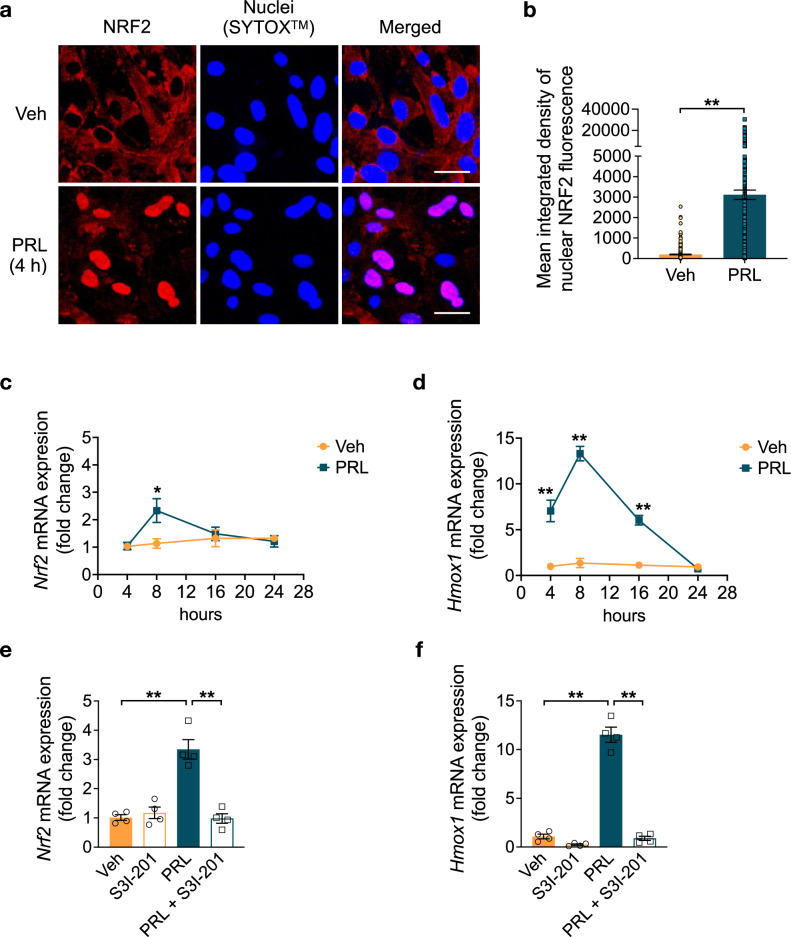
Effect of PRL on Nrf2 activation in rat astrocytes. **a** Representative NFE2 like bZIP transcription factor 2 (NRF2) immunostaining images of rat cortical astrocytes treated with 10 nM prolactin (PRL) for 4 h. Right panels are merged images of NRF2 (red, left panels) and the nuclear marker Sytox (blue, center panels). Scale bar 20 μm. **b** Quantification of nuclear NRF2 in control versus PRL-exposed rat cortical astrocytes. Data are means ± SEM of three independent experiments (n = 3). Rat cortical astrocytes were incubated in the absence or presence of 10 nM PRL for 4, 8, 16, or 24 h, and changes in mRNA levels of **c** *Nrf2* and **d** heme oxygenase 1 (*Hmox1)* were measured by quantitative RT-PCR. Data were initially normalized using the *Hprt* housekeeping gene as an internal control and then to the corresponding gene expression in the 4 h vehicle-treated group (Veh). Data in **c** and **d** are means ± SEM of four independent experiments (n = 4). Unpaired two-tailed Student’s t-test. Rat cortical astrocytes were incubated in the absence or presence of either 10 nM PRL, 100 μM STAT3 inhibitor S3I-201 or both for 8 h. mRNA levels of **e** *Nrf2* and **f** *Hmox1* were measured by quantitative RT-PCR. Data were initially normalized using the *Hprt* housekeeping gene as an internal control and then to the corresponding gene expression in the vehicle-treated group. Data in **e** and **f** are means ± SEM of four independent experiments (n = 4). One-way ANOVA followed by Tukey’s test. *p < 0.05, **p < 0.001 versus vehicle or indicated group

The NRF2-ARE (antioxidant response element) pathway is important for ROS detoxification that protects astrocytes from H_2_O_2_ toxicity [46]. Here, we investigated whether the NRF2-ARE signaling is involved in the PRL-mediated antioxidant mechanism. PRL treatment for 4 h elicited a significant accumulation of nuclear NRF2 in astrocytes (3389.22 ± 723.613 vs. 113.47 ± 73.43 A.U., *p* = 0.004) (Fig. 5a, b) and, thereby, the PRL-induced nuclear translocation of NRF2. Since NRF2 regulates its own expression [47], we determined the mRNA expression of *Nrf2* and its target gene, heme oxygenase 1 (*Hmox1*) at 4, 8, 16, and 24 h of PRL treatment. *Nrf2* mRNA increased two-fold after 8 h of incubation with PRL relative to control conditions (2.333 ± 0.4345 vs. 1.122 ± 0.1756; F_(7, 24)_ = 3.205, *p* = 0.026) (Fig. 5c). Notably, *Hmox1* mRNA levels were higher at 4, 8, and 16 h of incubation with PRL in comparison to control levels (4 h, 7.055 ± 1.175 vs. 1, *p* = 1.52 × 10^–5^; 8 h, 13.307 ± 0.787 vs. 1.358 ± 0.498, *p* = 1.56 × 10^–11^; 16 h, 6.037 ± 0.518 vs. 1.135 ± 0.259, *p* = 4.27 × 10^–5^; F_(7, 24)_ = 58.228), reaching a maximum of ~ 9.8-fold at 8 h of incubation, converging with the peak of *Nrf2* expression (Fig. 5d). Next, we evaluated whether STAT3 mediated the changes observed in *Nrf2* expression after exposure to PRL for 8 h. We found that the PRL-induced increase in *Nrf2* and *Hmox1* mRNA expression were abrogated when STAT3 signaling was inhibited by co-incubation with S3I-201 (Fig. 5e, f). These results show that PRL activates the NRF2 pathway via STAT3 signaling.

### PRL Increases Antioxidant Enzymatic Activity in Astrocytes

**Fig. 6:**
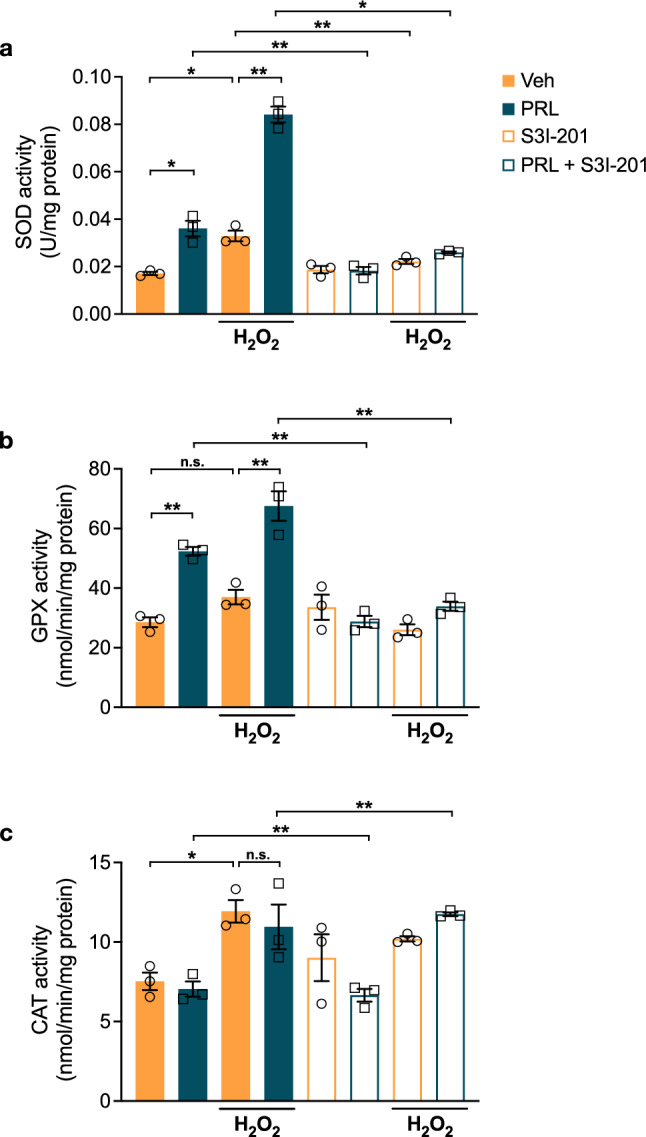
Effect of PRL on antioxidant enzymes activity in rat astrocytes. Rat cortical astrocytes were pre-incubated in the absence or presence of either 10 nM prolactin (PRL), 100 μM STAT3 inhibitor S3I-201 or both for 24 h, then 400 μM hydrogen peroxide (H_2_O_2_) or vehicle (Veh) was added and incubated for 3 h. **a** Superoxide dismutase (SOD), **b** glutathione peroxidase (GPX), and **c** catalase (CAT) activities were detected in samples of each treatment group containing equal amounts of protein using the corresponding commercial assay. Data in **a**, **b** and **c** are means ± SEM of three independent experiments (n = 3) carried out in duplicate. One-way ANOVA followed by Tukey’s test. *p < 0.05, **p < 0.001 versus indicated group; n.s., no significant difference

We then measured the enzymatic activity of SOD and GPX to determine whether the observed PRL-induced increase in the mRNA levels of these enzymes was accompanied by an increase in their activity. Treatment with PRL for 24 h resulted in a 2.1-fold increase in SOD activity (0.03604 ± 0.0033 vs. 0.01715 ± 0.0007 U/mg protein; F = _(7, 16)_ = 117.9; *p* = 0.000146; Fig. 6a) and ~ 1.84-fold increase in GPX activity (52.32 ± 1.413 vs. 28.46 ± 1.64 nmol/min/mg protein; F = _(7, 16)_ = 26.49; *p* = 0.00034; Fig. 6b). PRL further increased the stimulatory effect of H_2_O_2_ on SOD (0.08411 ± 0.0033 U/mg protein; *p* < 0.000001; Fig. 6a) and GPX (67.52 ± 4.919 nmol/min/mg protein; *p* = 0.0232; Fig. 6b) activities. Consistent with the mRNA levels, inhibition of STAT3 signaling blocked the effect of PRL alone or in combination with H_2_O_2_ on the activity of SOD and GPX (Fig. 6a, b). Neither PRL nor STAT3 inhibition affected the H_2_O_2_-induced increase in catalase activity, thus confirming that PRL does not regulate this enzyme (Fig. 6c). Taken together, these results indicate that PRL increases the amount of antioxidant enzymes available as well as their responsiveness against an oxidative insult.

### PRLR-Null Astrocytes are More Sensitive to H_2_O_2_ Toxicity

**Fig. 7:**
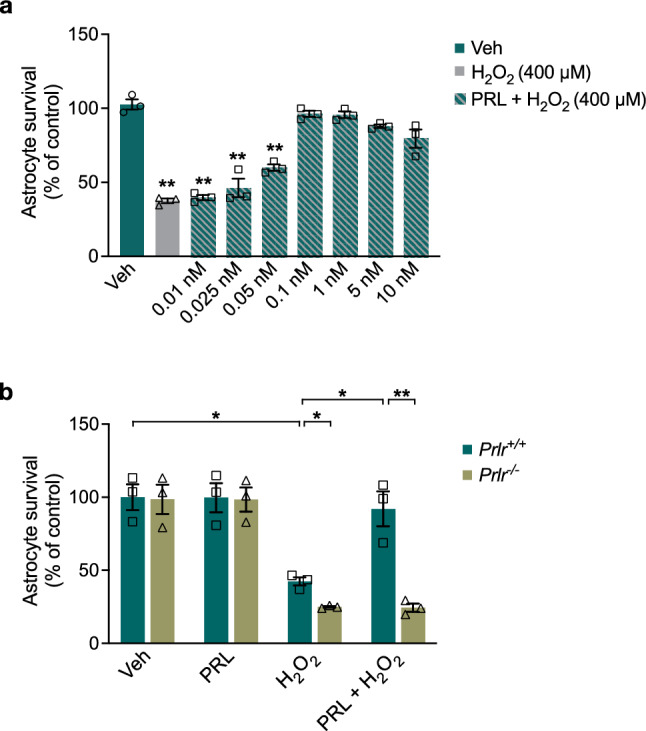
Effect of PRLR deficiency on H_2_O_2_-induced cell death in mouse astrocytes. **a** Mouse cortical astrocytes derived from wild-type (*Prlr*^+*/*+^) mice were pre-incubated for 24 h in the absence or presence of increasing concentrations of prolactin (PRL) (0.01–10 nM), then 400 μM hydrogen peroxide (H_2_O_2_) or vehicle (Veh) was added and incubated for 3 h. Data are means ± SEM of three independent experiments (n = 3) carried out in triplicate. **b** Mouse cortical astrocytes derived from wild-type (*Prlr*^+*/*+^) or null (*Prlr*^*−/−*^) mice were pre-incubated for 24 h in the absence or presence 1 nM PRL, then 400 μM H_2_O_2_ or vehicle was added and incubated for 3 h. Cell viability was quantified by MTT assay, and the results were normalized to PRLR wild-type control treated with vehicle. Data are means ± SEM of three independent experiments (n = 3) carried out in triplicate. One-way ANOVA followed by Tukey’s test. *p < 0.05, **p < 0.001 versus vehicle or indicated group

To determine whether the loss of PRLR affects the response of astrocytes to oxidative stress, we investigated the effect of H_2_O_2_ on astrocyte cultures from wild-type (*Prlr*^+*/*+^) or PRL receptor-null (*Prlr*^*−/−*^) mice. Astrocytes obtained from the brain cortex of newborn *Prlr*^+*/*+^ mice were more responsive to PRL than cortical astrocytes from rats. We observed that treatment for 24 h with 0.1 nM PRL, a concentration ten times lower than the previously established minimal effective concentration of 1 nM for PRL in rat astrocytes, completely prevented the reduction of cell viability induced by 400 μM H_2_O_2_. However, our results in Fig. 1c also demonstrate that 10 nM PRL offered stronger protection in rat astrocytes against even higher concentrations of H_2_O_2_ compared to 1 nM. Based on this observation, we chose to use a concentration of 1 nM PRL for mouse astrocytes, aiming for an equivalent level of protection observed with 10 nM in rat astrocytes (F_(8, 18)_ = 54.95; Fig. 7a). Astrocytes from *Prlr*^*−/−*^ mice did not exhibit any differences in cell viability in comparison with astrocytes from *Prlr*^+*/*+^ mice under control conditions. However, the incubation of astrocytes with 400 μM H_2_O_2_ for 3 h led to a 74.6% reduction of cell viability in astrocytes from *Prlr*^*−/−*^ mice (*p* = 0.000144), in contrast to the 57.6% observed with astrocytes from *Prlr*^+*/*+^ mice (*p* = 0.00199). As expected, the astrocytes from *Prlr*^*−/−*^ mice were resistant to the cytoprotective effect of 1 nM PRL (24.47 ± 2.769 vs. 92.01 ± 11.93%; *p* = 0.000378) (Fig. 7b). These results suggest a role for endogenous PRL/PRLR signaling in protecting astrocytes against H_2_O_2_-induced cell death.

### Effects of PRLR Deficiency on the H_2_O_2_-Induced Oxidative Damage and Antioxidant Capacity of Astrocytes

**Fig. 8:**
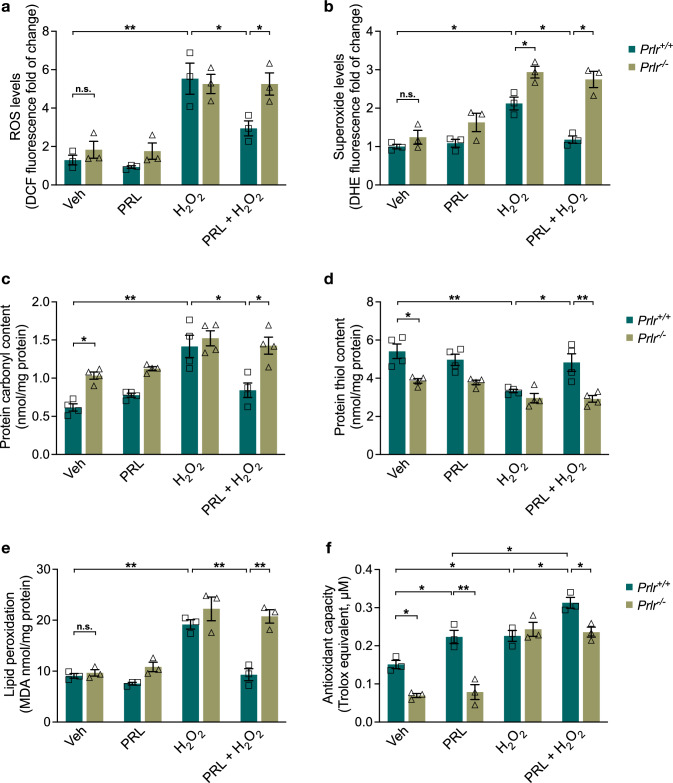
Effect of PRLR deficiency on H_2_O_2_-induced ROS and oxidative damage in wild-type and PRLR null mouse astrocytes. Mouse cortical astrocytes derived from wild-type (*Prlr*^+*/*+^) or null (*Prlr*^*−/−*^) mice were pre-incubated for 24 h in the absence or presence of 1 nM prolactin (PRL), then 400 μM hydrogen peroxide (H_2_O_2_) or vehicle (Veh) was added and incubated for 3 h. **a** Generation of reactive oxygen species (ROS) was quantified using 2′,7′-dichlorodihydrofluorescein diacetate (DCF-DA). Values are expressed as DCF fluorescence after 30 min incubation. Results are normalized to *Prlr*^+*/*+^ treated with vehicle. Data are means ± SEM of three independent experiments (n = 3) carried out in triplicate. **b** Generation of superoxide anion was measured using dihydroethidium (DHE). Values are expressed as DHE fluorescence after 30 min incubation. Results are normalized to *Prlr*^+*/*+^ treated with vehicle. Data are means ± SEM of three independent experiments (n = 3) carried out in triplicate. **c** Protein oxidation was estimated by measuring the protein carbonyl levels with the DNPH colorimetric assay. The concentration of the protein carbonyls was adjusted to the total protein concentration. Data are means ± SEM of four independent experiments (n = 4) carried out in duplicate. **d** Total sulfhydryl groups were measured by the reaction of free thiols in native proteins with DTNB. The concentration of the protein free thiols was adjusted to the total protein concentration. Data are means ± SEM of four independent experiments (n = 4) carried out in duplicate. **e** Lipid peroxidation was determined as increase in malondialdehyde (MDA), a thiobarbituric acid reactive substance (TBARS). The concentration of the MDA was adjusted to the total protein concentration. Data are means ± SEM of three independent experiments (n = 3) carried out in duplicate. **f** Antioxidant capacity was detected by ABTS assay. Data are means ± SEM of three independent experiments (n = 3) carried out in duplicate. One-way ANOVA followed by Tukey’s test; *p < 0.05, **p < 0.001 versus indicated group

We assessed the impact of PRLR ablation on the redox state under basal and H_2_O_2_-induced oxidative stress conditions in astrocytes. PRLR loss in astrocytes did not modify the physiological generation of ROS (Fig. 8a, b) but was associated with higher levels of superoxide than those observed in astrocytes from *Prlr*^+*/*+^ mice after H_2_O_2_ insult (2.94 ± 0.1549 vs. 2.122 ± 0.1676, F_(7,16)_ = 23.28. *p* = 0.03673, Fig. 8b). However, PRLR ablation in astrocytes resulted in increased basal protein oxidation, as revealed by a 1.67-fold increase in carbonyl content (1.034 ± 0.048 vs. 0.6162 ± 0.04737 nmol/mg protein, F_(7,24)_ = 15.46. *p* = 0.0375; Fig. 8c) and a 1.4-fold decrease in sulfhydryl group content (3.858 ± 0.1201 vs. 5.413 ± 0.3842 nmol/mg protein, F_(7,24)_ = 12.82. *p* = 0.00787; Fig. 8d), whereas basal lipid peroxidation did not change (Fig. 8e). Notably, *Prlr*^*−/−*^ astrocytes showed a 2.17-fold lower antioxidant capacity than *Prlr*^+*/*+^ astrocytes (0.06956 ± 0.0053 vs. 0.1510 ± 0.01075 Trolox equivalent μM; F_(7,16)_ = 35.95. *p* = 0.01748; Fig. 8f). In agreement with the findings in rat astrocytes, PRL prevented H_2_O_2_-induced oxidative damage in *Prlr*^+*/*+^ mouse astrocytes (Fig. 8a–e). PRL significantly reduced the H_2_O_2_-induced increase in ROS (2.946 ± 0.3928 vs. 5.534 ± 0.8109, *p* = 0.022; Fig. 8a), superoxide (1.182 ± 0.093 vs. 2.122 ± 0.1676, *p* = 0.01289; Fig. 8b), carbonyl group content (0.8414 ± 0.0952 vs. 1.417 ± 0.1471 nmol/mg protein, *p* = 0.001746; Fig. 8c), and lipid peroxidation (9.278 ± 1.178 vs. 19.15 ± 0.9214 MDA nmol/mg protein, *p* = 0.0004134; Fig. 8e) and prevented the H_2_O_2_-induced decrease in sulfhydryl levels (4.825 ± 0.4602 vs. 3.329 ± 0.0895 nmol/mg protein, *p* = 0.0114, Fig. 8d). In addition, PRL increased the total antioxidant capacity of *Prlr*^+*/*+^ astrocytes (0.2278 ± 0.01554 vs. 0.1510 ± 0.01075 Trolox equivalent μM, *p* = 0.02718). This effect was additive to that induced by the exposure to H_2_O_2_ (0.3134 ± 0.01374 Trolox equivalent μM, *p* = 0.01177). As expected, all the effects of PRL were absent in astrocytes lacking PRLR (Fig. 8a–e). Altogether, these data indicate a key role for PRL/PRLR endogenous signaling in regulating the redox state and the antioxidant response of astrocytes.

### Loss of PRLR Reduces Antioxidant Enzyme Activity in Astrocytes

**Fig. 9:**
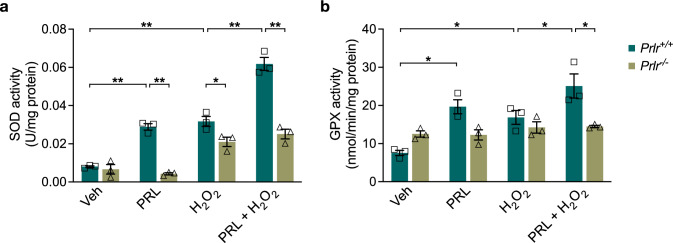
Antioxidant enzyme activities in wild-type and PRLR-null mouse astrocytes. Mouse cortical astrocytes derived from wild-type (*Prlr*^+*/*+^) or PRLR-null (*Prlr*^*−/−*^) mice were pre-incubated for 24 h in the absence or presence of 1 nM prolactin (PRL), then 400 μM hydrogen peroxide (H_2_O_2_) or vehicle (Veh) was added and incubated for 3 h. **a** Superoxide dismutase (SOD) and **b** glutathione peroxidase (GPX) activities were detected using the corresponding commercial assay. Data are means ± SEM of three independent experiments (n = 3). One-way ANOVA followed by Tukey’s test. *p < 0.05, **p < 0.001 versus indicated group; n.s., no significant difference

We analyzed SOD and GPX activity to evaluate whether the alterations in the redox state that favor an increased oxidative microenvironment in astrocytes from *Prlr*^*−/−*^ mice may be due to a deficiency in antioxidant enzyme activity. The basal activity of SOD (Fig. 9a) and GPX (Fig. 9b) was not changed in *Prlr*^*−/−*^ astrocytes in comparison with *Prlr*^+*/*+^ astrocytes. Despite having a higher superoxide generation upon treatment with H_2_O_2_, *Prlr*^*−/−*^ astrocytes did not exhibit lower SOD activity (Fig. 9a). Again, we confirmed that PRL significantly increased SOD (0.02887 ± 0.001674 vs. 0.0080 ± 0.00041 U/mg protein, F_(7, 16)_ = 79.95, *p* = 0.000058; Fig. 9a) and GPX (19.67 ± 1.837 vs. 7.536 ± 0.6958 nmol/min/mg protein, F_(7, 16)_ = 10.29, *p* = 0.001823; Fig. 9b) activity, and enhanced the H_2_O_2_-induced activity of both enzymes in *Prlr*^+*/*+^ astrocytes (SOD: 0.06177 ± 0.0033 U/mg protein; GPX: 25.11 ± 3.155 U/mg protein, Fig. 9a, b). As expected, these PRL effects were absent in astrocytes lacking PRLR signaling, demonstrating that PRLR loss impacts the antioxidant response of astrocytes.

## Discussion

**Fig. 10:**
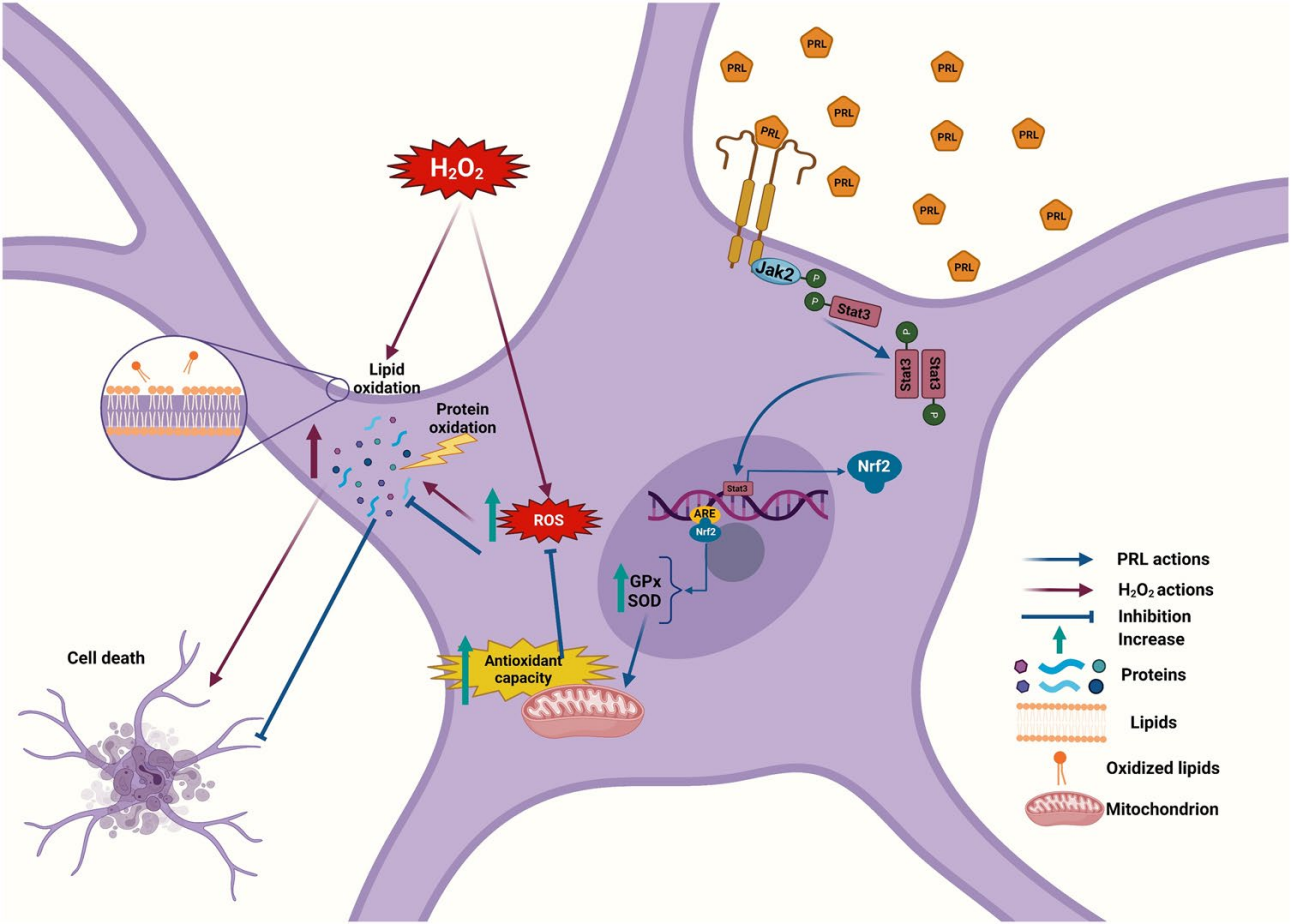
Schematic representation of the mechanism involved in the protective effect of PRL against H_2_O_2_-induced oxidative stress. PRL interaction with its receptor stimulates phosphorylation of transcription factor STAT3, which promotes the expression of enzymatic antioxidant systems (GPX, SOD) via NRF2, and thus increases the total antioxidant capacity. This cascade of events triggered by PRL reduces H_2_O_2_-induced ROS formation, protein oxidation, lipid peroxidation, and ultimately cell death. GPX, glutathione peroxidase; SOD, superoxide dismutase; ARE, antioxidant response element

Previous studies have shown that PRL plays a key role in controlling the survival of neurons [21, 48, 49]. The PRLR is present in neurons and astrocytes [14, 21]; however, the function of PRL in astrocytes remains obscure. To learn whether PRL exerts a protective effect on astrocytes, we used an in vitro approach based on pharmacological and loss-of-function strategies. Here we report a novel physiological role for PRL in the modulation of antioxidant systems in astroglial cells. We found that activation of the PRLR signaling cascade protects astrocytes challenged by H_2_O_2_-induced oxidative stress. PRL exerts this protective effect on astrocytes via the STAT3/NRF2 signaling pathway activation. This activation results in the increased expression and activity of antioxidant enzymes that, by enhancing the antioxidant capacity, reduce ROS production, protein oxidation, and lipid peroxidation, altogether preventing astrocytic cell death (Fig. 10).

Our study confirms that H_2_O_2_ exposure increases superoxide anion production, oxidative damage, and cell death in primary cortical astrocytes, consistent with previous reports [36, 37, 39–41, 50]. Notably, our findings demonstrate that PRL treatment significantly reduces superoxide anion accumulation and protects astrocytes from H_2_O_2_-induced cell death. This suggests a potential role for PRL in activating antioxidant defense enzymes, particularly superoxide dismutase (SOD). Prior research supports this possibility, as PRL has been shown to modulate SOD enzymes expression and activity in various tissues, including the CNS [51, 54, 55]. PRL induces an increase in the mRNA levels of cytosolic SOD1 and mitochondrial SOD2 enzymes in rat primary luteinized granulosa cells that can protect the corpus luteum against metabolic-related oxidative damage [51]. Furthermore, ageing rats with hyperprolactinemia exhibit an increase in SOD activity in the liver, thymus, and mammary gland [52, 53]. In the CNS, PRL up-regulates the protein levels of cytosolic SOD1 and mitochondrial SOD2 in primary hippocampal neurons. This up-regulation results in higher total SOD activity, which protects the neurons against glutamate excitotoxicity-induced oxidative damage [49]. However, PRL-mediated antioxidant effects have not been reported on glial cells. Here, we show that PRL up-regulated the mRNA expression of both cytosolic SOD1 and mitochondrial SOD2 in primary cortical astrocytes. It also markedly stimulated total SOD activity in astrocytes before and after exposure to H_2_O_2_, which correlated with a significant reduction of H_2_O_2_-induced superoxide anion accumulation. In contrast, astrocytes lacking PRLR showed reduced SOD activity and increased superoxide accumulation when exposed to H_2_O_2_. According to previous studies, astrocytes isolated from transgenic mice overexpressing SOD1 exhibit increased resistance to the superoxide generator menadione or ischemic-like oxygen and glucose deprivation [54, 55]. SOD2 overexpression in hippocampal astrocytes, on the other hand, reduces ROS production and oxidative damage [56]. Conversely, *Sod1* loss significantly increases Aβ oligomerization, plaque formation and oxidative damage in a mouse model of Alzheimer’s disease, and conditional knockout of *Sod2* in the brain is associated with increased levels of oxidative damage and lethality [57, 58]. Therefore, SOD activity is indispensable to cellular health and might be responsible, in great part, for the protective actions of PRL against H_2_O_2_-induced cell death.

Along with SODs, catalase, and GPX1 are also antioxidant defense enzymes in astrocytes [59]. Catalase is a highly efficient enzyme that reacts directly with H_2_O_2_ to form water and oxygen [60], while GPX1 is predominantly involved in the reduction of H_2_O_2_ and organic hydroperoxides to form water using reducing equivalents from GSH [61]. We have thus investigated the possible effect of PRL on catalase and GPX1 to protect primary cortical astrocytes against H_2_O_2_-induced cell death. Our data indicate that PRL has no effect on catalase expression or activity in astrocytes, independently of oxidant status. However, PRL up-regulates *Gpx1* mRNA expression and increase total GPX activity in the absence of oxidative stress. Moreover, astrocytes pretreated with PRL maintained markedly elevated GPX activity after exposure to 400 μM H_2_O_2_. This finding might seem contradictory because GPX is most active at lower H_2_O_2_ levels (10 μM) [62]. However, unlike other enzymes, GPX1 remains functional even when close to saturation at 100 μM H_2_O_2_. Therefore, the increased GPX1 abundance due to PRL treatment translates to enhanced cellular defense mechanisms, as the rate of enzymatic activity can be reliably estimated by the available GPX1 concentration. Accordingly, several studies show that GPX1 overexpression is followed by increased activity that enhances cell resistance to oxidant-induced damage [61, 63, 64]. Moreover, the fact that PRLR deficiency in astrocytes does not modify H_2_O_2_-induced GPX activity supports the notion that PRL’s effect on GPX activity is independent of the oxidant status. Additionally, we cannot discard the potential effect of PRL on GSH content in astrocytes, considering that GSH availability can impact GPX activity. In this regard, we previously demonstrated that PRL protects retinal pigment epithelium cells from H_2_O_2_-induced cell death by increasing GSH content [22]. In this study, PRL activation of the NRF2 pathway was observed, and NRF2 is known to regulate the expression of glutamate cysteine ligase, the rate-limiting enzyme in GSH synthesis in astrocytes [65, 66]. Hence, the role of PRL in the regulation of the GSH system in astrocytes remains to be elucidated.

Our findings highlight the critical role of STAT3 signaling in mediating PRL’s antioxidant effects in astrocytes. This aligns with previous research demonstrating STAT3 as a key regulator of astrocyte ROS detoxification and gene expression of antioxidant defenses [6]. Inhibition of STAT3 significantly reduced basal antioxidant capacity in our study, potentially due to its role in regulating GSH levels, a major contributor to cellular antioxidant capacity [67]. Supporting this notion, a previous study showed that conditional knockout of STAT3 or pharmacological inhibition of Jak2, which prevents STAT3 activation, results in a significant reduction of GSH levels in astrocytes [6]. Furthermore, we found that binding of PRL to its receptor activates the downstream JAK/STAT3 pathway in astrocytes, which is crucial to transducing the antioxidant effects of PRL and preventing H_2_O_2_-induced cell death. Our results showed that the STAT3 inhibitor S3I-201 completely abolished the protective effect of PRL, reversing its effects on ROS accumulation, protein oxidation, lipid peroxidation and, noticeably, decreased SOD and GPX activities, as well as the total antioxidant capacity in astrocytes. Moreover, the absence of the PRL receptor, which might decrease basal STAT3 activation, resulted in reduced thiol content and antioxidant capacity in basal conditions. This further supports a potential impact of PRL/STAT3 signaling on basal GSH levels, which remains to be confirmed.

Additionally, one plausible mechanism by which STAT3 mediates the antioxidant effects of PRL would be the increased expression of *Sod1*, *Sod2*, and *Gpx1* in astrocytes. To test this possibility, we inhibited STAT3 signaling in astrocytes treated with PRL and measured *Sod1*, *Sod2*, and *Gpx1* gene expression. Our results showed that STAT3 inhibition abolished PRL-induced *Sod1* and *Gpx1* expression, but not *Sod2* expression. These findings indicate that PRL could activate STAT3 signaling to promote *Sod1* and *Gpx1* gene expression in astrocytes. However, to date, the ability of STAT3 to regulate *Sod1* and *Gpx1* gene transcription has not been proven in reporter gene assays, and the lack of STAT3 in astrocytes does not modify SOD enzymes expression [6]. Therefore, it is also possible that these effects involve STAT3-mediated regulation of NRF2 by PRL in astrocytes. Along this line, the reduction or inhibition of STAT3 in cancer cells decreases NRF2 expression, its transcriptional activity, and antioxidant capacity [68–70]. Moreover, we found some pieces of evidence supporting both a direct and an indirect STAT3-NRF2 interaction. On the one hand, an online database (NRF2-ome) predicted the potential interplay of NRF2 with STAT3 based on domain-motif interactions [71]; which was confirmed in human breast cancer cells [72]. In these cancer cells, the active phosphorylated form of STAT3 interacts directly with the Neh1 and Neh3 domains of NFR2, and the concurrent binding of STAT3 and NRF2 to IL23A promoter accelerates breast cancer cells growth [72]. Evidence has confirmed that NRF2 mediates the normal expression of ARE-dependent genes in astrocytes [73] and that *Nrf2* knockdown is associated with the reduced expression of *Sod1*, *Sod2*, catalase, and *Gpx1* [74–76]. Here, we demonstrated that PRL stimulated the nuclear translocation of NRF2 and the mRNA levels of *Nrf2* and of the NRF2 target gene *Hmox1*. Further, PRL upregulation of these genes was abrogated by the inhibition of STAT3 signaling. Noticeably, the peak of *Nrf2* mRNA expression occurred eight hours after exposure to PRL, much earlier than the significant increase of *Sod1* and *Gpx1* expression observed 24 h after exposure to PRL. Subsequently, as previously reported [47], NRF2 may directly activate its own transcription, providing a positive feedback mechanism to amplify NRF2 effects. On the other hand, it has been recently shown an alternative mechanism for the functional interplay of STAT3 and NRF2 based on the binding of SOCS3 to KEAP1 [77]. Activated STAT3 induces the expression of certain SOCS genes, including *Socs3* that binds to phosphorylated JAK and its receptor to negatively regulate the JAK–STAT signaling pathway [78]. Meng et al. showed that SOCS3, derived from STAT3 activation, can directly bind to KEAP1 to prevent the degradation of NRF2, resulting in the activation of an NRF2-dependent transcriptional program in non-small cell lung cancer cells. This mechanism could be potentially involved in the interplay of STAT3 and NRF2 in response to PRL in astrocytes, considering that STAT3-dependent expression of SOCS3 has been observed in primary astrocytes [79, 80] and in response to PRL [81]. Thus, further investigation is needed.

Although STAT3 is known to upregulate *Sod2* expression in cortical and hippocampal neurons by binding to its promoter [82, 83], our findings suggest this pathway is not functional in astrocytes in response to PRL. The precise mechanism by which PRL upregulates *Sod2* expression in astrocytes remains to be elucidated. We found some evidence suggesting a potential role for the transcription factor NF-κB. The *Sod2* promoter contains a functional NF-κB binding site, and activation of NF-κB has been shown to increase *Sod2* expression in neurons and astrocytes [84, 85]. Notably, PRL activation of NF-κB has been observed in primary hippocampal neurons and mammary epithelial cells [86, 87]. Therefore, PRL-mediated activation of NF-κB could be a key factor involved in the upregulation of *Sod2* expression in astrocytes. Further investigation is necessary to confirm this hypothesis.

Open questions remain regarding the complete effects of PRL on astrocytes. Our data demonstrate that the lack of PRL receptor increased carbonyl group content under basal conditions. The precise reason for this accumulation remains unclear. Since ROS are constantly generated throughout cellular life and our data indicate that basal ROS production is not affected by the lack of PRL receptor, the accumulation of oxidized proteins could be due to two potential mechanisms: lower antioxidant capacity in the absence of PRL signaling, which is normally able to prevent protein oxidation, or decreased degradation of oxidized proteins, or a combination of both [88]. Further investigation is needed to elucidate the exact cause of this accumulation.

In conclusion, our study unveils the protective role of PRL against the oxidative stress-induced damage of astrocytes. The antioxidant effect of PRL might have both physiological and pathophysiological significance. Lactating rats exhibit reduced basal protein and lipid peroxidation in the hippocampus [89], suggesting that the high levels of circulating PRL observed in lactation may be involved in the protection against metabolically-induced oxidative damage in this reproductive period. Conversely, our previous work showed that the absence of PRL receptor in retinal pigmented epithelial cells of mice increased staining for the superoxide indicator DHE and reduced mRNA levels of catalase [22]. These findings suggest a potential link between PRL signaling and the regulation of oxidative stress in different cell types. On the other hand, because oxidative stress is one main factor for injury in CNS degenerative diseases and astrocytes mediate antioxidative processes in the brain [90], higher levels of PRL might delay glial and neuronal damage in pathological conditions associated to oxidative neurodegeneration. Clinical studies have shown that the concentration of PRL is elevated in the serum of patients with Parkinson’s disease [91] and in the cerebrospinal fluid of patients with Alzheimer’s disease with low Aβ1-42 levels [92]. Thereby, our data provides insights into the molecular basis of these diseases and the development of potential therapies to control them. However, our conclusions are based on in vitro data under an acute condition of oxidative stress. Whole animal studies and chronic conditions are needed to help solidify these findings.

## Data Availability

The data that support the findings of this study are available from the corresponding author upon reasonable request.
